# The Effect of Hydrolyzed Insect Meals in Sea Trout Fingerling (*Salmo trutta* m. *trutta*) Diets on Growth Performance, Microbiota and Biochemical Blood Parameters

**DOI:** 10.3390/ani10061031

**Published:** 2020-06-13

**Authors:** Zuzanna Mikołajczak, Mateusz Rawski, Jan Mazurkiewicz, Bartosz Kierończyk, Damian Józefiak

**Affiliations:** 1 Department of Animal Nutrition, Poznań University of Life Sciences, Wołyńska 33, 60-637 Poznań, Poland; bartosz.kieronczyk@up.poznan.pl; 2 Institute of Zoology, Division of Inland Fisheries and Aquaculture, Poznań University of Life Sciences, Wojska Polskiego 71c, 60-625 Poznań, Poland; mateusz.rawski@up.poznan.pl (M.R.); jan.mazurkiewicz@up.poznan.pl (J.M.)

**Keywords:** *Tenebrio molitor*, *Zophobas morio*, hydrolyzed insect meals, fish nutrition, fish health, blood biochemistry, microbiota, growth performance, feed efficiency

## Abstract

**Simple Summary:**

The replacement of fishmeal by environmentally sustainable alternative meals has been one of the targets in aquaculture in recent decades. A number of factors support the use of insect meals, as a group of products characterized by high crude protein and crude fat content, in fish nutrition. Insects are readily accepted by a number of fish species, and they are part of the natural diet of omnivorous and carnivorous species. The present study was conducted to evaluate the effects of hydrolyzed *Tenebrio molitor* and *Zophobas morio* meals as a partial replacement for fishmeal in sea trout (*Salmo trutta* m. *trutta*) diets on growth performance, feed utilization, organosomatic indices, serum biochemistry, gut histology, and microbiota. In the present study, insect meals inclusion did not cause any adverse impacts on growth performance, feed utilization or gut histomorphology. However, an effect on the organosomatic indices, serum biochemistry, and microbiota was observed. In conclusion, hydrolyzed *T*. *molitor* and *Z*. *morio* meals seem to be promising alternative protein sources for sea trout nutrition.

**Abstract:**

The present study is the first introduction of hydrolyzed superworm meal in sea trout nutrition. It was conducted to evaluate the effects of inclusion in the diet of hydrolyzed insect meals as a partial replacement for fishmeal on growth performance, feed utilization, organosomatic indices, serum biochemical parameters, gut histomorphology, and microbiota composition of sea trout (*Salmo trutta* m. *trutta*). The experiment was performed on 225 sea trout fingerlings distributed into three groups (3 tanks/treatment, 25 fish/tank). The control diet was fishmeal-based. In the experimental groups, 10% of hydrolyzed mealworm (TMD) and superworm (ZMD) meals were included. The protein efficiency ratio was lower in the TMD and ZMD. Higher organosomatic indices and liver lipid contents were found in the group fed ZMD. The ZMD increased levels of aspartate aminotransferase, and decreased levels of alkaline phosphatase. The *Aeromonas* spp. and *Enterococcus* spp. populations decreased in the ZMD. The concentrations of the *Carnobacterium* spp. decreased in the ZMD and TMD, as did that of the *Lactobacillus* group in the TMD. In conclusion, insect meals may be an alternative protein source in sea trout nutrition, as they yield satisfying growth performance and have the capability to modulate biochemical blood parameters and microbiota composition.

## 1. Introduction

The replacement of fishmeal (FM) by environmentally sustainable alternative protein sources has been one of the targets of aquaculture in recent decades [1]. Plant meals have been used as the main alternative; however, insect meals have recently emerged as one of the most promising components of fish nutrition [2,3,4]. This is caused by their high nutritive value—protein and fat content, anti-pathogenic and anti-inflammatory properties connected with antimicrobial peptides, and lauric acid and chitin presence—as well as their environmental sustainability due to their taking part in a circular economy and wide presence in the natural diets of many fish species [5,6]. Nevertheless, in the available literature, information about the effect of insect meals on growth performance, microbiota of the gastrointestinal tract, and blood biochemical parameters in salmonid fish is still scarce, and most of the research is focused only on salmon and rainbow trout [7,8,9,10,11].

One of the key factors that has caused increased interest in research for alternative feed materials is the growing price of FM and its harmful effects on the environment [12,13]. However, many alternatives can lead to secondary, adverse health effects—nutritional disorders and metabolic disturbances may be caused by the use of dietary plant meal, i.e., enteritis in the distal intestine, hypertrophic mucus production, a reduction in reproductive rates, high mortality or growth depression [14,15]. Another important source of protein—animal byproducts, such as blood meal, meat and bone meal, as well as feather meal—is associated with legislative issues and consumer intolerance; moreover, it may contain antinutritional factors, i.e., indigestible pepsin or high levels of crude ash [16] or cause amino acid imbalance in the diet due to high levels of proline or glycine and low tryptophan and tyrosine content [17].

Compared with other salmonids, sea trout (*Salmo trutta* m. *trutta*) tolerate higher water temperatures, and may play a crucial role in aquaculture under the pressure of the progressing global warming [18]. In the wild, they consume a wide spectrum of terrestrial and aquatic insects, which makes insect meals natural and environmentally sustainable feed components for the species [19,20]. The dietary inclusion of up to 25% insect meal does not affect growth performance in most fish species [9,21,22,23,24,25,26,27], and some research conducted on turbot and seabass [28,29] demonstrated an improvement in performance and digestibility. Moreover, some substances present in insects, such as chitin and antimicrobial peptides (AMPs), may play an important role in immune system modulation [30,31] and the stabilization of the homeostasis between animal and host gut microbiota [32]. However, it must be emphasized that processing—such as drying, fat extraction or enzymatic hydrolysis [33], as well as rearing methods and technologies, i.e., usage of different organic waste substrates [3,34,35], rearing period length and environmental conditions [36]—can also improve the nutritional value of insect meals.

In the available literature, data about the use of insect meals in sea trout diets are scarce and they can be found in only one published study described recently by our team [37]. Moreover, information on the microbiota of this species is very limited. The effects of insects on blood plasma parameters and immunological responses have been studied only in salmon [38]. However, the presence of insect meals may positively affect the microbiota composition and improve the gastrointestinal health of these animals [7,31].

Considering all the aspects mentioned above, this research aimed to evaluate the impact of mealworm (*Tenebrio molitor*) and superworm (*Zophobas morio*) larval meals hydrolyzed by bacterial enzymes and used as partial replacements for fishmeal on the blood immune responses and the microbiota of the gastrointestinal tract of sea trout compared to their growth performance and the feed efficiency.

## 2. Materials and Methods

### 2.1. Insect Meals

The insects were purchased from a commercial supplier (HiProMine S.A., Robakowo, Poland). To obtain full-fat meals, the larvae were oven-dried at 50 °C for 24 h and finely ground. The chemical composition and amino acid profile of the meals were determined for diet formulation (Table 1 and Table 2).

### 2.2. Preparation of Insect Meal Hydrolysates

Two commercial proteases were used to hydrolyze the dried larvae meals in two subsequent steps. The full-fat larvae meals were ground and mixed using distilled water at a ratio of 4:1 (w:v) to achieve a consistency suitable for enzyme hydrolysis. Initially, the diluted bacterial (*Bacillus amyloliquefaciens*) endopeptidase enzyme Corolase^®^ 7090 (AB Enzymes GmbH, Darmstadt, Germany) was added to the meals at a concentration of 1.5 g·kg^−1^ of protein, and the mixture was heated for five hours at 50 °C, according to the manufacturer’s instructions. Next, 0.75 g·kg^−1^ of the fungal protease enzyme Flavourzyme^®^ (endopeptidase and exopeptidase from *Aspergillus oryzae*; supplied by Novozymes A/S, Denmark) was added to the mixture, which was homogenized and hydrolyzed for three hours. The hydrolyzed meals were kept at 4 °C until diet preparation.

### 2.3. Diet Formulation and Preparation

A control diet (CON) and two experimental diets were formulated (Table 3). The control diet was based on previous results described by our research team and literature [37,39]. A 10% hydrolyzed insect meal inclusion was fixed for both the experimental diets. Hydrolyzed mealworm (TMD) and superworm (ZMD) meals were used. The isonitrogenous (490 g·kg^−1^ of crude protein) and isoenergetic (22.5 MJ·kg^−1^) diets were manufactured at the Feed Production Technology and Aquaculture Experimental Station in Muchocin, Poland. In the feed production process, all the dried ingredients were mixed with hydrolyzed insect biomass in a batch-type ribbon blender (WBN-150, WAMGROUP S.p.A., Ponte Motta/Cavezzo, MO, Italy). The mixture was then passed through a semi-industrial single-screw extruder (Metalchem S-60, Gliwice, Poland) at 110 °C to obtain pellets with 1.5-mm and 2.5-mm diameters. After extrusion, the pellets were dried in an airflow drier for 24 h at 40 °C. After drying, fish oil was added onto the mildly heated pellets. The diets were packed in plastic bags and stored at −18 °C until use.

### 2.4. Fish and Feeding Trial

All animal handling protocols and methods complied with the recommendations of Directive 2010/63/EU of the European Parliament and the council of 22 September 2010 on the protection of animals used for scientific purposes, the Polish law of 15 January 2015 on the protection of animals used for scientific purposes (Dz.U.2015 poz. 266), and the good practices and recommendations of the National Ethics Committee for Animal Experiments and the Local Ethics Committee for Animal Experiments of Poznań University of Life Sciences [40]. The trial was performed in the Division of Inland Fisheries and Aquaculture, which, as a unit of the Faculty of Veterinary Medicine and Animal Science of Poznań University of Life Sciences, is certified for animal experiments (approved unit no. 0091) by the National Ethics Committee for Animal Experiments (based on authorization by the Ministry of Science and Higher Education).

Sea trout fingerlings were transported from the Feed Production Technology and Aquaculture Experimental Station in Muchocin, Poland, to the Division of Inland Fisheries and Aquaculture laboratory. Firstly, fish were acclimated for seven days. After that time, on the 1st day of the experimental period, 225 fish with an average body weight of 5.08 ± 0.9 g were weighed individually and distributed randomly into nine tanks (25 fish in each). The fiberglass tanks used had 60 L capacities, which were supplied with 2 L min^−1^ water from the reservoir in an open-flow system. The water parameters were recorded daily. The temperature was 14.7 ± 0.6 °C, the dissolved oxygen was constant at 7.5 ± 0.3 mg L^−1^, and the photoperiod was maintained at 16:8 (light:dark) throughout the experiment.

The trial lasted eight weeks, and, during this time, the animals were fed by automatic band feeders (12 h discharge time, FIAP Fishtechnik GmbH., Ursensollen, Germany). During the experiment, the animals were weighed and counted every two weeks to adjust the feed intake ratio, and the growth and feed efficiency parameters were recorded. The feed ratio was based on a feeding chart designed for Atlantic salmon, taking into consideration the average body weight of the fingerlings and the water temperature [39]. The fish mortality was monitored daily.

### 2.5. Sample Collection and Organosomatic Indices

At the beginning of the trial, 200 g of euthanized fish biomass was collected and preserved at 20 °C for body composition analysis. Fish biomass was weighted for each tank, and animals used for somatic indices calculations and other analysis were weighed and measured individually. The growth performance test was performed in triplicate—3 tanks per treatment— and each tank was considered as experimental unit (n = 3/treatment—representing 25 fish/tank). The fish were euthanized using an overdose of MS-222 [41] and decapitated for dissection, sampling and further analysis. Blood sampling was performed postmortem. Blood samples were collected from the caudal veins of nine fish per tank with nonheparinized 1-ml syringes. The samples were pooled, with 3 fish/sample, which was the experimental unit (3 samples/tank, n = 9/treatment). The samples were kept in a refrigerator for 30 min to allow the formation of a clot. The serum was separated by centrifugation at 4 °C and 3500 RPM for 15 min (Hettich Zentrifugen, Tuttlingen, Germany). The serum samples were stored at −82 °C for further analysis. The viscera and liver weights were recorded and used for the calculation of the organosomatic indices. The livers from five fish per tank were removed for the determination of liver glycogen and triglycerides (n = 15/treatment). The whole-body proximate compositions were determined for all the sampled fish. All the samples were kept at −82 °C until they were used for further analysis.

### 2.6. Analysis of the Biological Material

The raw materials, experimental diets, and fish carcasses were analyzed according to the Association of Official Agricultural Chemists (AOAC) [42] methodology for dry matter (934.01), crude protein (976.05), ether extract (920.39), crude ash (920.153) and crude fiber (985.29). The proximate amino acid and fatty acid compositions of the hydrolyzed insect meals and experimental diets were analyzed according to AOAC (2005) [42] procedures at an accredited laboratory (J.S. Hamilton S. A, Gdynia, Poland). The amino acids were determined using high-performance liquid chromatography (HPLC) with an Automatic Amino Acid Analyzer AAA 400 (Ingos Ltd., Prague, Czech Republic). The fatty acid composition of the hydrolyzed insect meals and experimental diets was analyzed according to the method described in EN-ISO 12966-1:2015-01 (European standard). The gross energy content was analyzed according to the ISO 9831 method using an adiabatic bomb calorimeter (KL 12 Mn, Precyzja-Bit PPHU, Bydgoszcz, Poland) standardized with benzoic acid. The chitin composition of the insect meals was determined using the method described by Soon et al. (2018) [43].

### 2.7. Blood Serum Immunology

The blood serum immunology was assessed according to the methods described by Kołodziejski et al. (2018) [44]. For the assays of serum concentrations of total protein (TP), albumin, glucose, triglycerides, and total cholesterol (TC), commercial assay kits (Pointe Scientific, Canton, MI, USA) were used. A liquid assay reagent set (catalog number: ALT, A7526; AST, A7560; ALP, A7516, Pointe Scientific, Canton, MI, USA) was used to determine the enzymatic activities of alanine aminotransferase (ALT), aspartate transaminase (AST), and alkaline phosphatase (ALP).

The free fatty acids (FFA) content as well as the glycogen levels were measured by enzymatic assay kits from Wako Diagnostics (China); the methodology described by Kołodziejski et al. (2017) [45] was used for these analyses. Five liver samples from each tank were weighed and immediately placed into tubes containing KOH (30%). Then, the liver samples were boiled for 15 min and cooled. Next, ethanol (98%) and distilled water were added into the tubes. The samples were then centrifuged at 3500 rpm for 30 min, and the supernatants were discarded. A citrate buffer (pH 4.4) with enzyme glucoamylase (activity: 12,000 U/L) was added to the tubes, and the samples were incubated for 2 h at 55 °C to hydrolyze glycogen into glucose. To determine the glucose concentrations in the solutions, a colorimetric enzyme assay was used (glucose oxidase, Pointe Scientific, Canton, MI, USA), and these values were used to calculate the concentrations of glycogen in the liver tissues.

The levels of immunoglobulin M (IgM) and lysozyme (LZM) in the serum were measured using double antibody sandwich ELISA kits (Sunred Biotechnology, Shanghai, China).

### 2.8. Liver Triglycerides

The liver triglyceride levels were determined according to methods described by Kołodziejski et al. (2018) [44]. The lipids in the liver samples were extracted by a modified Folch method [46], which includes bead mill disruption of the tissue material with a Tissue Lyzer II (Qiagen, Germany). After the extraction of the lipids, the concentration of the triglycerides was determined by a colorimetric enzyme assay kit (glycerol phosphate oxidase, Pointe Scientific, Canton, MI, USA).

### 2.9. Gut Histomorphology

Samples of the anterior portion of the intestines of nine fish per treatment were collected and submerged in Bouin’s solution (Merck) until analysis, which was performed according to methods described by Sobolewska et al. (2017) [47]. The samples were dehydrated, cleared and embedded in paraffin blocks. Formed blocks were cut on a rotary microtome (Thermo Shandon, Chadwick Road, Astmoor, Runcorn, Cheshire, United Kingdom) into slices of 10-μm thickness [48]. The slices were placed on microscope slides coated with ovo albumin with an addition of glycerol. The samples were analyzed using an AnMN-800 F microscope (OPTA-TECH, Warsaw, Poland) equipped with an Opta-View camera for recording microscopic images. MultiScan v. 18.03 microscope imaging software (Computer Scanning Systems II Ltd., Warsaw, Poland) was used to measure villus height and width and muscular thickness.

### 2.10. Microbial Community Analysis by Fluorescent In Situ Hybridization (FISH)

The gastrointestinal tract digesta taken from the gut of 12 fish per tank was sampled, and digesta was pooled into 3 samples per tank (the experimental unit was a sample representing 4 fish n = 9/treatment). The digesta was immediately frozen and stored at −82 °C. The samples were prepared and observed following the protocols described by Rawski et al. (2016; 2018) [49,50]. The oligonucleotide probes used for this study are shown in Table 4. The samples were visualized using a Carl Zeiss Microscope Axio Imager M2. The numbers of detected bacteria are expressed in colony-forming units/g of digesta (CFU mL^−1^) and were calculated according to the equation of Józefiak et al. 2019 [7] given below:

(1) $$\log CFU/g=\log(N\times (\frac{WA}{PA})\times (\frac{Sweight+Dweight}{Sweight})\times (\frac{1000}{Svolume}))$$

where N is the number of visible bacterial cells, *WA* is the working area of the filter, *PA* is the picture area, *Sweight* is the sample weight, *Dweight* is the dilution factor weight, and *Svolume* is the volume of the sample pipetted onto the filter.

### 2.11. Statistical Analysis

SAS software was used to analyze the data. To determine the normality of the data distribution and equality of the variances, Kolmogorov–Smirnov and Levene’s tests were used. One-way ANOVA was used, and, if there were significant differences among the treatments, further analysis was performed by a corrected Duncan’s post hoc test. The data are presented as the mean ± standard error of the mean (SEM). The statistical significance level was set at *p* < 0.05.

The analysis of variance was conducted according to the following general model:

(2) $$Y_{i}=\mu +\alpha_{i}+\delta_{\mathrm{ij}}$$

where Y_i_ is the observed dependent variable, μ is the overall mean, α_i_ is the effect of the diet, and δ_ij_ is the random error.

## 3. Results

### 3.1. Amino Acid and Fatty Acid Composition

The amino acid (AA) composition of the experimental diets is presented in Table 5. Compared with those in the TMD and ZMD treatments, the control diet exhibited a similar ratio between IAA and DAA. The contents of EPA and DHA were relatively low in both types of meals. In the case of experimental diets, the inclusion of insect meals affected the fatty acid profile, especially the content of n-3 PUFAs. Compared with that in the control diet, the n-3/n-6 fatty acid ratios decreased in the experimental diets containing insects (Table 6).

### 3.2. Growth Performance and Nutrient Utilization

At the end of the experimental period, the growth performance parameters (final body weight, body weight gain (BWG), and specific growth rate (SGR)) were not affected by the inclusion of the insect meals (Table 7). Among the feed efficiency parameters, the daily feed intake (DIR), feed conversion ratio (FCR), and protein production value (PPV) were not different among the treatment groups; only the protein efficiency ratio (PER) significantly decreased in TMD and ZMD in comparison to CON (*p* = 0.029).

The sea trout fingerlings fed with ZMD exhibited higher hepatosomatic index (HSI) (*p* < 0.001) and viscerosomatic index (VSI) (*p* = 0.010) values than those in the CON and TMD treatments. These values were related to the significant increase in lipid content in the liver in the ZMD treatment (*p* = 0.004). However, the liver glycogen and whole-body composition parameters were not affected by the inclusion of the insect meals (Table 8).

### 3.3. Blood Serum Immunology

Significant differences were found in the serum analysis (Table 9). The aspartate aminotransferase (AST) value increased in the fish provided feed supplemented with ZMD (*p* = 0.002). ZMD meal reduced the concentration of alkaline phosphatase (ALP) compared to the levels of this enzyme in the CON and TMD treatments (*p* < 0.001). The concentration of triglycerides was significantly lower in the TMD treatment than in the CON treatment (*p* = 0.034); however, no differences were observed between the ZMD and other treatments. Additionally, the albumin content as well as the total cholesterol in the blood serum increased in those fish that consumed the insect meals (*p* = 0.010; *p* < 0.001, respectively). In the case of alanine aminotransferase (ALT), total protein content, free fatty acids, glucose, immunoglobulin M and lysozyme, there were no significant differences observed among the treatments.

### 3.4. Gut Histomorphology

The anterior part of the gastrointestinal tract did not exhibit any significant differences in villus height, villus width, or villus area among the sea trout fed the insect meals or the control diet. The muscular layer thickness was also not affected by insect meals inclusion (Table 10).

### 3.5. Microbial Community Analysis by Fluorescent In-Situ Hybridization (FISH)

The inclusion of ZMD meal significantly decreased the concentrations of *Aeromonas* spp., *Enterococcus* spp. and *Carnobacterium* spp. (*p* = 0.037, *p* = 0.017, and *p* = 0.001, respectively); the concentration of *Carnobacterium* spp. also decreased in the fish in the TMD treatment. The inclusion of TMD meal significantly reduced the concentration of the *Lactobacillus* group (*p* = 0.025). The total number of bacteria and *Bacillus* spp. did not exhibit any significant differences among the treatments (Table 11).

## 4. Discussion

Currently, insect meals are promoted as natural alternative protein sources for fish nutrition. Although insects are a source of high-quality protein, they also provide antimicrobial peptides (AMPs) and chitin, which are considered health promoters in animal nutrition [15,31,55]. Chitin and its derivative products have been demonstrated to be beneficial to fish nutrition and health [56,57]. AMPs are the main contributors to microbiological homeostasis in insects due to their activity against potentially pathogenic bacteria, fungi, parasites and viruses [58]. However, it should be emphasized that FM replacement by insect meals creates the need to supplement certain amino acids, especially methionine (Table 1).

The present study represents the first introduction of hydrolyzed *Z*. *morio* meal to sea trout diets. The results are characterized by a high survival rate and satisfactory growth performance and feed utilization parameters [37,59]. The positive effect of hydrolyzed fish protein was previously proven by a significant reduction in vertebral anomalies [60]. Moreover, protein hydrolysate was previously observed as a positive factor in growth and disease resistance [61]. The main mode of action that is used to explain this effect is the fact that hydrolyzed meals may contain low-molecular-weight compounds. These compounds may be related to more effective absorption, which results in improved growth performance and feed utilization [62]. In the present study, there were no differences in the final body weight, BWG, SGR, DIR, FCR, PPV or survival rate among the treatment groups. However, the protein efficiency ratio was reduced significantly in those fish fed insect meals. These results are in agreement with those of Stadtlander et al. [8], who showed that the inclusion of black soldier fly (BSF, *Hermetia illucens*) meal in a rainbow trout diet yielded results similar to those of a control diet in terms of growth and feed conversion. However, protein utilization was decreased in the BSF group. These differences may be due to the crude protein bonding to the chitin [63], which causes an overestimation of its content in the raw materials and the diet, causing a reduction in the PER values. It is important to emphasize that the nitrogen-to-protein conversion factor (Kp) of 6.25 generally used for proteins leads to an overestimation of protein content in insects because of the presence of nonprotein nitrogen (NPN) sources, such as chitin, nucleic acids, or phospholipids. To avoid this overestimation, Kp values of 4.76 for the protein content in whole insects and 5.60 for the protein extracts should be used [64]. The results of the present study are in agreement with the findings of Hoffmann et al. [37], in which no effect on average total length or body mass, SGR, FCR, or PER was observed throughout the experiment. A significant effect of insect meals on survival rate was not observed in either study; however, in the present study, the survival rate was numerically higher and close to 100%.

Among the organosomatic indices, the HSI and VSI of the fish fed with ZMD were significantly higher than those of the TMD and CON groups. Moreover, these differences were related to the higher liver lipid levels found in the fish in the ZMD treatment. This can be explained by the presence of higher levels of palmitic acid (PA) in the diet with ZMD meal than in the other diets, as this caused the enlargement of the liver due to the accumulation of fat in this organ. A similar increase in the HSI was observed in Japanese sea bass (*Lateolabrax japonicus*) fed diets with relatively high levels of PA [65]. Hoffmann et al. (2020) [37] have shown a reduction in VSI values in sea trout fed hydrolyzed and full-fat mealworm meals compared to those fed the control diet. However, in the same study, the inclusion of *T*. *molitor* full-fat meal and the use of a diet with *T*. *molitor* hydrolyzed with a 1.0% mixture of enzymes did not lead to differences in the HSI values compared to those generated by a fishmeal-based diet [37]. According to Huang et al. (2016) [66], higher lipid levels in diets lead to fat storage in the visceral cavity and liver of fish; however, this accumulation may also depend on the fatty acid composition of the diet. This may be the reason for the observed differences in the effects of dietary inclusions on organosomatic indices between *T*. *molitor* and *Z. morio*; specifically, this pattern may be due to differences in PA and saturated fatty acid (SFA) content, which was higher in the ZMD treatment group. What is more, the ratio between n-3 and n-6 fatty acids is reported as a potential modulate factor of the biochemical composition of fish liver and its structure as well as metabolism [25,67,68]. The reduction in n-3 fatty acid composition in insect diets creates an imbalance in the n-3/n-6 ratio which increases the lipid deposits in the liver. The higher level of n-6 in fish products can negatively affect human health due to the pro-inflammatory properties of this acid [69]. However, the effect of the diet on the chemical composition of fish, and, indirectly, on human health, should be considered in further studies.

In terms of the hematological parameters of the serum, there were no significant differences in the ALT concentration. However, the AST analyses showed higher values in the ZMD treatment group. These two aminotransferases are potential biomarkers of liver health [70]. According to this information, any degradation of the liver will elevate the concentration of this enzyme. The inclusion of BSF meal in the diets of Atlantic salmon led to a decrease in the ALT concentration, while the AST concentration was not affected by diet [11]. In the case of birds, insect meal inclusion showed no effect on these aminotransferase concentrations in barbary partridges (*Alectoris barbara*) [71] or laying hens [72]. In contrast, the inclusion of mealworm meal in broiler chicken diets increased the concentrations of both parameters; however, the possibility of liver damage was excluded by analyses of other enzymes [73]. Perhaps the enlargement observed in our study represents only the excessive fat accumulation due to the high content of PA mentioned previously. These results suggest that further analyses are needed to correctly describe the effects of insect use on liver physiology. According to the literature, ALP is present in the membrane of almost all animal cells, and its activity is commonly related to cellular damage [74,75]. The decrease in this enzyme in the ZMD group may be related to cellular homeostasis. The reduction in this compound is related to an improvement in health status in birds [44]. In contrast, in children, higher levels of ALP are associated with bone growth [76]; therefore, our findings may indicate that the level of ALP is correlated with numeric differences in growth rate in the TMD and CON groups, which was suggested previously by Lemieux et al. (1999) [77] in a study conducted on Atlantic cod (*Gadus morhua*). The total protein content, which is considered an indicator of nutritional status [78], was not affected by insect meal inclusion. According to Panettieri et al. (2020) [79] variations in the total protein content in the blood serum of fish can be a response to a number of physiological changes, i.e., tissue injury or destruction, differences in blood volume and plasma hydration and organism response to stress conditions. However, those effects were not observed in the present study. The albumin levels were significantly higher in the TMD and ZMD groups than in the control. The proteins in blood serum mainly consist of albumin and globulin. It was reported that, in adult Atlantic salmon, albumin constituted approximately 40% of the serum protein values [80]. Previous studies have tried to establish the normal values of blood parameters in healthy Atlantic salmon, and, according to this information, the albumin levels oscillated between 18 and 24 g·L^−1^; however, these values vary seasonally, and data were available for adult fish only [81]. In common carp (*Cyprinus carpio*) exposed to heavy metals at lethal and sublethal concentrations, albumin levels may be significantly decreased to meet the immediate energy demand of toxic stress [80]. In birds, albumin has been shown to be a source of the amino acids necessary for tissue protein synthesis [82]. The fish fed both insect meals showed significantly higher cholesterol levels than those in the CON group, while the inclusion of TM meal led to a decrease in triglycerides. According to the current literature, the inclusion of BSF meal in Atlantic salmon, rainbow trout, and Jian carp (*Cyprinus carpio var*. Jian) diets did not have an impact on total cholesterol or triglyceride content [83,84,85]. On the other hand, a number of papers have reported that the addition of insect meal can reduce the concentrations of total cholesterol as well as triglycerides in the blood serum of various fish species [29,30,86,87]. These results are mainly explained as a positive effect of dietary chitin, which has the ability to bind bile acids and free fatty acids [31]. In addition, it was suggested that blood cholesterol may play an important role in the immune defense system [88]; however, in the present study, the increase in total cholesterol concentration was not related to higher lysozyme activity, which is an important nonspecific immune system factor in fish. Insect meals inclusion did not affect the content of IgM, which contributes to innate and adaptive immunity in fish [89]. It has been observed that mealworm meal supplementation reinforces the innate and adapted immune responses of yellow catfish (*Pelteobagrus fulvidraco*) [90]. In general, the results obtained from the hematological analyses in this study allow us to conclude that the inclusion of insect meals in sea trout diets did not negatively affect blood parameters; in particular, the fish growth performance and survival rate were not affected among any of the groups.

The hydrolyzed insect meals did not affect the gut histomorphology (i.e., the villus height, width and area and muscular thickness), which has a crucial role in nutrient absorption and gut health. The use of mealworm and BSF meal in Siberian sturgeon (*Acipenser baerii*) diets did not affect villus height [91]. Moreover, the mucosal thickness was lower in fish fed with added BSF meal; in contrast, mealworm diets increased the thickness of the muscular layer. However, in a study carried out on rainbow trout (*Oncorhynchus mykiss*), the villus height decreased with mealworm and tropical house cricket (*Gryllodes sigillatus*) inclusion, while an increase in villus height was observed with the addition of the Turkestan cockroach (*Blatta lateralis*) [7]. In this study, the mucosal thicknesses were lower and higher in the tropical house cricket and Turkestan cockroach treatment groups, respectively, than in the control group. Moreover, the inclusion of BSF meal in the diet decreased the prevalence of steatosis in the proximal intestine of Atlantic salmon [38]. This variety of results arises mainly from the wide range of fish species used in the experiments as well as the use of different insect species and their level of inclusion in the feed. Moreover, the technology used in the production of the diets, the drying process, fat extraction, etc., as well as for storage, may affect the properties of insect meals as well as their effects on gut microstructures. Thus, the focus should be placed on species-specific solutions as well as on processing technologies for further studies and gut health assessments.

The abovementioned findings suggest that a crucial role is played by the GIT microbiome. The present study shows a lack of effects of insect supplementation on the total number of bacteria, which is in agreement with the findings of research carried out by Bruni et al. (2018) [92] on rainbow trout. This result indicates that the use of partially defatted BSF meal did not affect the amount of digesta-associated bacterial communities; however, it did increase the number of mucosa-associated bacteria. This study showed that the inclusion of insect meals also had no influence on the concentration of *Bacillus* spp. This genus of bacteria is well known, due to its probiotic properties and production of secondary metabolites, such as acetic acid, lactic acid and bacteriocins, and may contribute to potentially improving fish health [93,94]. In terms of insect meal use, it has been reported that the inclusion of mealworms at a concentration of 50% in fish diets reduced *Bacillus* spp. in gilt-head bream (*Sparus aurata*) and brown trout [95]. In contrast, the inclusion of mealworm as well as BSF meal led to an increase in *Bacillus* spp. in the case of Siberian sturgeon [91]. The inclusion of *Z*. *morio* meal significantly decreased the concentration of *Carnobacterium* spp., but, in the case of the *T*. *molitor* meal, a reduction in *Carnobacterium* spp. as well as the *Lactobacillus* group was observed. Both *Carnobacterium* spp. and the *Lactobacillus* group are lactic acid bacteria (LAB), which produce inhibitory substances against fish pathogens [96]. The described effect can potentially provide an increased chance for pathogen proliferation and further microbiota imbalance. However, despite those changes, a significant decrease in the concentration of *Aeromonas* spp. and *Enterococcus* spp was observed in the ZM group. The reduction in *Aeromonas* spp. is a positive change, as this genus includes pathogenic and opportunistic bacteria, such as *Aeromonas hydrophila* and *Aeromonas salmonicida*, that may produce cytotoxins, enterotoxins and endotoxins, negatively affecting intestinal barrier functions [97,98]. The decrease in *Enterococcus* spp. can also be seen as a positive effect of ZM meal inclusion. Despite the fact that a number of bacteria belonging to this genus may be used as probiotics due to their antimicrobial properties, many of them are virulent, and this can lead to the invasion of host tissue and displacement through epithelial cells [99]. The obtained results are in opposition to the findings of an experiment on *A*. *baerii*, which showed a decrease in *Carnobacterium* spp., the *Lactobacillus* group, *Aeromonas* spp., and *Enterococcus* spp. in a control treatment fishmeal [91]. However, in a study that included BSF, mealworm, tropical house cricket, and Turkestan cockroach meal in rainbow trout diets, the concentration of LAB from *Lactobacillus* sp./*Enterococcus* sp. was lower in all the groups fed with insects than in the control group, which was fed fishmeal. In the same experiment, the number of *Enterobacteriaceae* bacteria increased as a result of *T*. *molitor* inclusion. Moreover, according to Osimani et al. (2019) [100] the microbiota of black soldier fly may have been influenced by the feeding substrates used during the rearing process of insects. The microbiological composition of insects can be connected with the rearing methods and used substrates, which affect the microbiological value of insect meals due to secondary metabolites of bacteria and bacteriocin expression. Those differences indirectly affected the bacterial community of the gastrointestinal tract of zebrafish (*Danio rerio*) [100]. 

All described results can be explained by the presence of chitin, which is a component of the exoskeleton of insects, shellfish, fungi, molds, and protozoa. It is considered as a factor modulating the microbiome of the gastrointestinal tract [101]. It has been shown that a 5% addition of chitin to the diet of *Salmo salar* affected individual bacterial groups by decreasing the populations of *Bacillus* spp., *Lactobacillus* spp., *Pseudomonas* spp., and *Staphylococcus* spp. [102]. The antimicrobial properties of shrimp chitin and chitosan against many pathogenic microorganisms, i.e., *Escherichia coli*, *Pseudomonas aeruginosa*, *Listeria monocytogenes*, and *Staphylococcus aureus*, have been observed [103], which suggest the potential of usage chitin and its derivatives as prebiotic or immunostimulants [104,105]. The effect of dietary chitin on the composition of the gastrointestinal tract of *Gadus morhua* was also demonstrated by Zhou et al. (2013) [106]. However, it has to be emphasized that the presence of chitin in the diet can reduce feed intake and digestibility as well as nutrient absorption [26,28] due to possible intestinal inflammation [25,30]. The second explanation for the differences in microbiological composition among the groups may be the presence of AMPs. AMPs can be classified into four groups (α-helical peptides, cysteine-rich peptides, proline-rich peptides, and glycine-rich proteins), and, depending on the group they belong to, these peptides may be effective against a wide spectrum of potentially pathogenic bacteria species, i.e., *S*. *aureus*, *E*. *coli*, *L*. *monocytogenes*, and *Salmonella typhimurium* [58].

## 5. Conclusions

The use of hydrolyzed insect meals did not affect the growth performance or feed efficiency parameters, including final body weight, BWG, SGR, DIR, FCR and PPV. The effects of ZMD meal were found in the HSI, VSI and liver lipids. Effects of ZMD and TMD on serum biochemistry were observed, which are key points for further studies due to the limited information about hematological parameters in fish. In terms of intestinal histomorphology, there were no aberrations or structural changes, which can be considered an advantage of using insects for sea trout nutrition. The ZMD and TMD treatments decreased the concentrations of groups of bacteria key for fish health, including the *Lactobacillus* group, *Carnobacterium* spp., *Aeromonas* spp., and *Enterococcus* spp., which provides a need for further complex analyzes of insect meal effects on fish microbiome and health. In general, hydrolyzed insect meals appear to be a promising alternative protein source for sea trout nutrition.

## Figures and Tables

**Table 1:** Amino acid profiles of hydrolyzed insect larvae meals (% of crude protein) compared to the fishmeal used in the study.

Item	Insect Larvae Meals		
Amino Acids	TM ^1^	ZM ^2^	FM ^3^
Indispensable amino acids (IAA)			
Arginine	5.02	4.50	5.91
Histidine	3.21	3.04	2.13
Isoleucine	4.77	4.67	4.79
Leucine	8.34	7.61	7.99
Lysine	5.64	5.62	7.99
Methionine	1.62	1.46	3.11
Phenylalanine	4.28	3.98	3.99
Threonine	5.21	4.97	4.39
Valine	7.40	6.94	5.77
Tryptophan	1.00	1.16	1.08
Dispensable amino acids (DAA)			
Alanine	9.09	8.32	6.79
Aspartic acid	8.28	8.19	9.70
Cysteine	0.83	0.75	0.90
Glycine	5.96	5.21	6.56
Glutamic acid	13.87	13.79	14.57
Proiline	7.17	6.13	4.30
Serina	5.09	4.48	4.45
Tyrosine	6.85	6.61	3.10
IAA/DAA	0.81	0.82	0.94

^1^ TM, enzyme-hydrolyzed mealworm meal; ^2^ ZM, enzyme-hydrolyzed superworm meal; ^3^ FM, fishmeal (Skagen, Denmark).

**Table 2:** Fatty acid composition of the hydrolyzed insect meal (g·kg^−1^ dry matter).

Item	Insect Larvae Meals	
Fatty Acids	TM ^1^	ZM ^2^
C14:0	29	9
C16:0	192	306
C18:0	35	91
Saturated fatty acids (SFA)	259	421
C16:1n7	19	6
C18:1n9	409	300
Monounsaturated fatty acids (MUFA)	435	315
C18:2n-6 (LA)	281	239
C20:4n-6 (ARA)	<0.1	<0.1
Polyunsaturated fatty acids (PUFA n-6)	281	239
C18:3n-3 (LNA)	13	9
C18:4n-3	<0.1 ^3^	<0.1
C20:5n-3 (EPA)	<0.1	<0.1
C22:5n-3	<0.1	<0.1
C22:6n-3 (DHA)	<0.1	<0.1
Polyunsaturated fatty acids (PUFA n-3)	13	9
n-3/n-6	0.05	0.04
PUFA/SFA	1.14	0.60
C14:0	29	9
C16:0	192	306
C18:0	35	91

^1^ TM, enzyme-hydrolyzed mealworm meal; ^2^ ZM, enzyme-hydrolyzed superworm meal; ^3^ <0.1 = fatty acids detected at lower amounts than 1 g·kg^−1^ of sample.

**Table 3:** Formulation and analyzed chemical composition of the experimental diets.

Ingredients (g·kg^−1^)	Diets		
	CON ^1^	TMD ^2^	ZMD ^3^
Fishmeal ^4^	250	145	140
Mealworm meal ^5^	-	100	-
Superworm meal ^6^	-	-	100
Soybean meal ^7^	100	100	100
Wheat flour	219	220	226
Corn gluten	150	150	150
Blood meal ^8^	70	100	100
Brewer yeast	35	35	35
Fish oil	164	143	140
Dicalcium phosphate	7.2	0.8	2.1
Premix ^9^	1.5	1.5	1.5
DL-Methionine	1.2	2.2	2.4
L-Lysine HCL	1.1	1.8	2.0
L-Threonine	0.6	0.6	0.7
**Proximate analysis (g·kg^−1^ DM)**			
Dry matter	930	937	935
Crude protein	480	511	498
Crude lipid	163	146	153
Ash	65	54	51
Crude fiber	17	17	17
Chitin ^10^	0	9.3	4.8
NFE ^11^	350	338	350
Gross energy (MJ·kg^−1^)	22.18	22.77	22.55

^1^ CON—fishmeal diet (control); ^2^ TMD—enzyme-hydrolyzed mealworm diet; ^3^ ZMD—enzyme-hydrolyzed superworm diet; ^4^ Skagen, Denmark (crude protein: 71.4%); ^5^ Mealworm meal, HiProMine S.A., Poland (dry matter: 95.58%, crude protein: 47.0%, crude lipid: 29.6%); ^6^ Superworm meal, HiProMine S.A., Poland (dry matter: 96.32%, crude protein: 49.3%, crude lipid: 33.6%); ^7^ Solvent-extracted 45% crude protein, 1.8% crude lipid; ^8^ Spray-dried 90% protein, APC Europe, Spain; ^9^ Polfamix BASF Poland Ltd. (Kutno, Poland) (g·kg^−1^ ): vitamin A, 1 000,000 IU; vitamin D_3_, 200,000 IU; vitamin E, 1.5 g; vitamin K, 0.2 g; vitamin B_1_, 0.05 g; vitamin B_2_, 0.4 g; vitamin B_12_, 0.001 g; nicotinic acid, 2.5 g; D-calcium pantothenate, 1.0 g; choline chloride, 7.5 g; folic acid, 0.1 g; methionine, 150.0 g; lysine, 150.0 g; Fe, 2.5 g; Mn, 6.5 g; Cu, 0.8 g; Co, 0.04 g; Zn, 4.0 g; J, 0.008 g; carrier > 1000.0 g.; ^10^ Calculated amount from chitin analysis of insect meals; ^11^ Nitrogen-free extract = 1000 − (crude protein + ether extract + crude fiber + ash).

**Table 4:** Oligonucleoide probes used in fluorescent in-situ hybridization with fish fed three diets.

Probe	Group of Bacteria	Sequence 5’–3’	References
Aer66	*Aeromonas* spp.	CTA CTT TCC CGC TGC CGC	[51]
Bmy843	*Bacillus* spp.	CTT CAG CAC TCA GGT TCG	[52]
CAR193	*Carnobacterium* spp.	AGC CAC CTT TCC TTC AAG	[51]
Enfm93	*Enterococcus* spp.	CCG GAA AAA GAG GAG TGG C	[53]
Lab722	*Lactobacillus* group	YCA CCG CTA CAC ATG RAG TTC CAC T	[54]

**Table 5:** Amino acid profiles of experimental diets (g·kg^−1^ dry matter).

Amino Acids	Diets		
	CON ^1^	TMD ^2^	ZMD ^3^
Indispensable amino acids (IAA)			
Arginine	21.7	20.3	21.5
Histidine	12.9	13.9	14.5
Isoleucine	15.3	13.9	14.7
Leucine	36.1	36.8	39.8
Lysine	27.2	24.9	27.1
Methionine	12.5	11.7	12.6
Phenylalanine	21.4	21.8	23.2
Threonine	18.6	18.2	15.8
Valine	24.0	24.5	26.5
Tryptophan	4.3	4.5	4.6
Dispensable amino acids (DAA)			
Alanine	23.1	24.0	25.7
Aspartic acid	32.6	33.0	36.0
Cysteine	4.9	5.8	5.3
Glycine	21.6	20.4	21.4
Glutamic acid	93.2	89.9	99.0
Proline	31.9	31.8	34.1
Serine	19.5	20.5	21.1
Tyrosine	12.2	13.2	14.0
IAA/DAA	0.81	0.80	0.78

^1^ CON—fishmeal diet (control); ^2^ TMD—enzyme-hydrolyzed mealworm diet; ^3^ ZMD—enzyme-hydrolyzed superworm diet.

**Table 6:** Fatty acid profiles of experimental diets (g·kg^−1^ dry matter).

Fatty Acids	Diets		
	CON ^1^	TMD ^2^	ZMD ^3^
C14:0	38	39	35
C16:0	135	146	176
C18:0	27	31	42
Saturated fatty acids (SFA)	211	223	261
C16:1n7	43	39	35
C18:1n9	276	300	303
Monounsaturated fatty acids (MUFA)	492	500	479
C18:2n-6 (LA)	97	108	106
C20:4n-6 (ARA)	5	8	7
Polyunsaturated fatty acids (PUFA n-6)	114	123	120
C18:3n-3 (LNA)	27	23	21
C18:4n-3	16	15	14
C20:5n-3 (EPA)	38	31	28
C22:5n-3	5	8	7
C22:6n-3 (DHA)	60	46	42
Polyunsaturated fatty acids (PUFA n-3)	162	139	113
n-3/n-6	1.43	1.13	0.94
PUFA/SFA	1.31	1.17	0.89
C14:0	38	39	35
C16:0	135	146	176
C18:0	27	31	42

^1^ CON—fishmeal diet (control); ^2^ TMD—enzyme-hydrolyzed mealworm diet; ^3^ ZMD—enzyme-hydrolyzed superworm diet.

**Table 7:** Growth performance and feed utilization of sea trout fed with experimental diets.

Item	Diets			
	CON ^1^	TMD ^2^	ZMD ^3^	*p*-Value
Initial body weight (g)	5.75 ± 0.04	5.84 ± 0.03	5.85 ± 0.12	0.639
Final body weight (g)	21.20 ± 0.51	20.97 ± 0.72	20.60 ± 0.20	0.729
BWG (g) ^4^	15.47 ± 0.50	15.10 ± 0.69	14.77 ± 0.09	0.632
SGR (%/day) ^5^	2.33 ± 0.04	2.28 ± 0.06	2.25 ± 0.02	0.435
DIR (%/day) ^6^	1.39 ± 0.02	1.44 ± 0.04	1.48 ± 0.03	0.184
FCR ^7^	0.99 ± 0.02	1.04 ± 0.04	1.07 ± 0.02	0.200
PER ^8^	2.10 ± 0.04 ^a^	1.88 ± 0.07 ^b^	1.87 ± 0.04 ^b^	0.029
PPV (%) ^9^	32.51 ± 0.81	28.82 ± 2.08	28.58 ± 0.53	0.140
Survival (%) ^10^	100 ± 0.00	99 ± 1.33	99 ± 1.33	0.630

^1^ CON—fishmeal diet (control); ^2^ TMD—enzyme-hydrolyzed mealworm diet; ^3^ ZMD—enzyme-hydrolyzed superworm diet; values in the same row having different superscript letters are significantly different at *p* < 0.05, (n = 3); ^4^ Body weight gain (BWG) = [(final body weight—initial body weight (g))/initial body weight, g] × 100; ^5^ Specific growth rate (SGR) = [(ln final body weight (g)—ln initial body weight (g))/number of days] × 100; ^6^ Daily intake rate (DIR) = [(feed intake (g)/total weight (g))/number of days] × 100; ^7^ Feed conversion ratio (FCR) = total feed supplied (g DM)/weight gain (g); ^8^ Protein efficiency ratio (PER) = [weight gain (g)]/total protein fed (g DM); ^9^ Protein production value (PPV) = [protein retention in fish (g DM)/total protein fed (g DM) × 100; ^10^ Survival = [total number of fish harvested/total number of fish stocked] × 100; values in the same row having different superscript letters are significantly different at *p* < 0.05 (n = 3); 3.3. Organosomatic indices and body composition.

**Table 8:** Organosomatic indices and whole-body composition (% of wet weight) of sea trout fed with experimental diets.

Item	Diets			
	CON ^1^	TMD ^2^	ZMD ^3^	*p*-Value
Organosomatic indices (%)				
HIS ^4^	1.45 ± 0.04 ^b^	1.44 ± 0.05 ^b^	1.78 ± 0.07 ^a^	<0.001
VSI ^5^	7.78 ± 0.17 ^b^	7.94 ± 0.21 ^b^	8.62 ± 0.22 ^a^	0.010
Liver energy reserves (%)				
Liver lipid	4.05 ± 0.18 ^b^	4.12 ± 0.14 ^b^	4.96 ± 0.26 ^a^	0.004
Liver glycogen	1.76 ± 0.07	1.8 ± 0.05	1.71 ± 0.06	0.549
Whole-body composition (%)				
Moisture	76.16 ± 0.16	76.05 ± 0.73	76.00 ± 0.35	0.973
Crude protein	15.45 ± 0.06	15.40 ± 0.47	15.28 ± 0.11	0.914
Crude lipid	5.39 ± 0.13	5.42 ± 0.27	5.47 ± 0.16	0.955
Crude ash	2.39 ± 0.07	2.51 ± 0.07	2.60 ± 0.06	0.150

^1^ CON—fishmeal diet (control); ^2^ TMD—enzyme-hydrolyzed mealworm diet; ^3^ ZMD—enzyme-hydrolyzed superworm diet; values in the same row having different superscript letters are significantly different at *p* < 0.05; ^4^ Hepatosomatic index (HSI) = [(liver weight (g)/body weight (g)] × 100; ^5^ Viscerosomatic index (VSI) = [(viscera weight (g)/body weight (g)] × 100; values in the same row having different superscript letters are significantly different at *p* < 0.05 (n = 15).

**Table 9:** Serum biochemistry values of sea trout fed with experimental diets.

Item	Diets			
	CON ^1^	TMD ^2^	ZMD ^3^	*p*-Value
Alanine aminotransferase (U L^−1^)	4.56 ± 1.12	4.54 ± 0.83	3.92 ± 0.42	0.616
Aspartate aminotransferase (U L^−1^)	32.29 ± 3.07 ^b^	30.86 ± 4.07 ^b^	55.46 ± 7.25 ^a^	0.002
Alkaline phosphatase (U L^−1^)	90.49 ± 3.91 ^a^	79.80 ± 3.65 ^a^	65.81 ± 3.52 ^b^	<0.001
Total protein (g L^−1^)	55.6 ± 1.1	57.2 ± 1.1	54.4 ± 0.4	0.097
Albumin (g L^−1^)	25.7 ± 0.2 ^b^	27.3 ± 0.4 ^a^	27.4 ± 0.6 ^a^	0.010
Triglycerides (mg dL^−1^)	493.19 ± 15.84 ^a^	384.25 ± 33.82 ^b^	412.04 ± 29.61 ^ab^	0.034
Total cholesterol (mg dL^−1^)	290.41 ± 11.08 ^b^	365.12 ± 13.37 ^a^	342.86 ± 6.54 ^a^	<0.001
Free fatty acids (mmol L^−1^)	0.60 ± 0.01	0.60 ± 0.02	0.58 ± 0.01	0.522
Glucose (mg dL^−1^)	87.56 ± 3.07	91.17 ± 3.59	88.33 ± 2.89	0.702
Immunoglobulin M (mg mL^−1^)	0.40 ± 0.03	0.37 ± 0.03	0.45 ± 0.06	0.419
Lysozyme (µg mL^−1^)	15.48 ± 1.07	14.11 ± 0.95	15.05 ± 0.99	0.615

^1^ CON—fishmeal diet (control); ^2^ TMD—enzyme-hydrolyzed mealworm diet; ^3^ ZMD—enzyme-hydrolyzed superworm diet; values in the same row having different superscript letters are significantly different at *p* < 0.05 (n = 9).

**Table 10:** Histomorphology of the anterior portion of the gut of sea trout fed with experimental diets.

Item	Diets			
	CON ^1^	TMD ^2^	ZMD ^3^	*p*-Value
Villus height	275.83 ± 14.58	326.20 ± 14.58	287.55 ± 14.58	0.056
Villus width	85.00 ± 4.38	93.15 ± 4.38	97.18 ± 4.38	0.156
Villus area	4438.84 ± 372.8	4798.02 ± 372.8	5345.44 ± 395.5	0.245
Muscular layer thickness	80.13 ± 4.23	73.38 ± 4.23	82.70 ± 4.23	0.293

^1^ CON—fishmeal diet (control); ^2^ TMD—enzyme-hydrolyzed mealworm diet; ^3^ ZMD—enzyme-hydrolyzed superworm diet; Values in the same row having different superscript letters are significantly different at *p* < 0.05 (n = 9).

**Table 11:** Microbiology results of the digesta of sea trout fed with experimental diets.

Target	Diets			
	CON ^1^	TMD ^2^	ZMD ^3^	*p*-Value
Total number of bacteria	9.49	9.52	9.54	0.576
*Aeromonas* spp.	9.34 ^a^	9.36 ^a^	9.24 ^b^	0.037
*Carnobacterium* spp.	9.34 ^a^	9.17 ^b^	9.10 ^b^	0.001
*Enterococcus* spp.	9.29 ^a^	9.29 ^a^	9.15 ^b^	0.017
*Lactobacillus* group	9.38 ^a^	9.18 ^b^	9.23 ^ab^	0.025
*Bacillus* spp.	9.13	9.12	9.19	0.508

^1^ CON—fishmeal diet (control); ^2^ TMD—enzyme-hydrolyzed mealworm diet; ^3^ ZMD—enzyme-hydrolyzed superworm diet; Values in the same row having different superscript letters are significantly different at *p* < 0.05 (n = 9).

## References

[1] Huntington T.C., Hasan M.R. (2009). Fish as feed inputs for aquaculture-practices, sustainability and implications: A global sinthesis. Fish as Feed for Aquaculture: Practices, Sustainability and Implications.

[2] Engle C.R., D’Abramo L., Ponniah A.G., Slater M. (2017). Global aquaculture 2050. J. World Aquacult. Soc..

[3] Zarantoniello M., Zimbelli A., Randazzo B., Compagni M.D., Truzzi C., Antonucci M., Riolo P., Loreto N., Osimani A., Milanović V. (2020). Black Soldier Fly (*Hermetia illucens*) reared on roasted coffee by-product and Schizochytrium sp. as a sustainable terrestrial ingredient for aquafeeds production. Aquaculture.

[4] Gasco L., Acuti G., Bani P., Dalle Zotte A., Danieli P.P., De Angelis A., Fortina R., Marino R., Parisi G., Piccolo G. (2020). Insect and fish by-products as sustainable alternatives to conventional animal proteins in animal nutrition. Ital. J. Anim. Sci..

[5] Nogales-Mérida S., Gobbi P., Józefiak D., Mazurkiewicz J., Dudek K., Rawski M., Kierończyk B., Józefiak A. (2018). Insect meals in fish nutrition. Rev. Aquac..

[6] Gahukar R.T. (2016). Edible Insects Farming: Efficiency and Impact on Family Livelihood, Food Security, and Environment Compared With Livestock and Crops. Insects as Sustainable Food Ingredients.

[7] Józefiak A., Nogales-Mérida S., Mikołajczak Z., Rawski M., Kierończyk B., Mazurkiewicz J. (2019). The Utilization of Full-Fat Insect Meal in Rainbow Trout (*Oncorhynchus mykiss*) Nutrition: The Effects on Growth Performance, Intestinal Microbiota and Gastrointestinal Tract Histomorphology. Ann. Anim. Sci..

[8] Stadtlander T., Stamer A., Buser A., Wohlfahrt J., Leiber F., Sandrock C. (2017). *Hermetia illucens* meal as fish meal replacement for rainbow trout on farm. J. Insects Food Feed.

[9] Lock E.R., Arsiwalla T., Waagbø R. (2016). Insect larvae meal as an alternative source of nutrients in the diet of Atlantic salmon (*Salmo salar*) postsmolt. Aquac. Nutr..

[10] Li Y., Kortner T.M., Chikwati E.M., Belghit I., Lock E.-J., Krogdahl Å. (2020). Total replacement of fish meal with black soldier fly (*Hermetia illucens*) larvae meal does not compromise the gut health of Atlantic salmon (*Salmo salar*). Aquaculture.

[11] Belghit I., Liland N.S., Waagbø R., Biancarosa I., Pelusio N., Li Y., Krogdahl Å., Lock E.J. (2018). Potential of insect-based diets for Atlantic salmon (*Salmo salar*). Aquaculture.

[12] Hardy R.W. (2010). Utilization of plant proteins in fish diets: Effects of global demand and supplies of fishmeal. Aquac. Res..

[13] Olsen R.L., Hasan M.R. (2012). A limited supply of fishmeal: Impact on future increases in global aquaculture production. Trends Food Sci. Technol..

[14] Francis G., Makkar H.P.S., Becker K. (2001). Antinutritional factors present in plant-derived alternate fish feed ingredients and their effects in fish. Aquaculture.

[15] Oliva-Teles A. (2012). Nutrition and health of aquaculture fish. J. Fish Dis..

[16] Meeker D.L., Hamiltom C.R. (2006). An Overview of the Rendering Industry in: Essential Rendering—All about the Animal by-Products Industry.

[17] Jayathilakan K., Sultana K., Radhakrishna K., Bawa A.S. (2012). Utilization of byproducts and waste materials from meat, poultry and fish processing industries: A review. J. Food Sci. Technol..

[18] Vandeputte M., Labbé L. (2012). Cultured Aquatic Species Information Programme. *Salmo trutta*. Cultured Aquatic Species Fact Sheets.

[19] Skrodenyte V., Sruoga A., Butkauskas D., Skrupskelis K. (2008). Phylogenetic analysis of intestinal bacteria of freshwater salmon *Salmo salar* and sea trout *Salmo trutta trutta* and diet. Fish. Sci..

[20] Elliott J.M. (1967). The Food of Trout (*Salmo trutta*) in a Dartmoor Stream. J. Appl. Ecol..

[21] Dietz C., Liebert F. (2018). Does graded substitution of soy protein concentrate by an insect meal respond on growth and N-utilization in Nile tilapia (*Oreochromis niloticus*)?. Aquac. Rep..

[22] Katya K., Borsra M.Z.S., Ganesan D., Kuppusamy G., Herriman M., Salter A., Ali S.A. (2017). Efficacy of insect larval meal to replace fish meal in juvenile barramundi, *Lates calcarifer* reared in freshwater. Int. Aquat. Res..

[23] Gasco L., Henry M., Piccolo G., Marono S., Gai F., Renna M., Lussiana C., Antonopoulou E., Mola P., Chatzifotis S. (2016). *Tenebrio molitor* meal in diets for European sea bass ( *Dicentrarchus labrax* L.) juveniles. Anim. Feed Sci. Technol..

[24] Zarantoniello M., Randazzo B., Truzzi C., Giorgini E., Marcellucci C., Vargas-Abúndez J.A., Zimbelli A., Annibaldi A., Parisi G., Tulli F. (2019). A six-months study on Black Soldier Fly (*Hermetia illucens*) based diets in zebrafish. Sci. Rep..

[25] Vargas-Abúndez A.J., Randazzo B., Foddai M., Sanchini L., Truzzi C., Giorgini E., Gasco L., Olivotto I. (2019). Insect meal based diets for clownfish: Biometric, histological, spectroscopic, biochemical and molecular implications. Aquaculture.

[26] Zarantoniello M., Bruni L., Randazzo B., Vargas A., Gioacchini G., Truzzi C., Annibaldi A., Riolo P., Parisi G., Cardinaletti G. (2018). Partial Dietary Inclusion of *Hermetia illucens* (Black Soldier Fly) Full-Fat Prepupae in Zebrafish Feed: Biometric, Histological, Biochemical, and Molecular Implications. Zebrafish.

[27] Vargas A., Randazzo B., Riolo P., Truzzi C., Gioacchini G., Giorgini E., Loreto N., Ruschioni S., Zarantoniello M., Antonucci M. (2018). Rearing Zebrafish on Black Soldier Fly (*Hermetia illucens*): Biometric, Histological, Spectroscopic, Biochemical, and Molecular Implications. Zebrafish.

[28] Kroeckel S., Harjes A.G.E., Roth I., Katz H., Wuertz S., Susenbeth A., Schulz C. (2012). When a turbot catches a fly: Evaluation of a pre-pupae meal of the Black Soldier Fly (*Hermetia illucens*) as fish meal substitute—Growth performance and chitin degradation in juvenile turbot (*Psetta maxima*). Aquaculture.

[29] Magalhães R., Sánchez-López A., Leal R.S., Martínez-Llorens S., Oliva-Teles A., Peres H. (2017). Black soldier fly (*Hermetia illucens*) pre-pupae meal as a fish meal replacement in diets for European seabass (*Dicentrarchus labrax*). Aquaculture.

[30] Li S., Ji H., Zhang B., Zhou J., Yu H. (2017). Defatted black soldier fly (*Hermetia illucens*) larvae meal in diets for juvenile Jian carp (*Cyprinus carpio* var. Jian): Growth performance, antioxidant enzyme activities, digestive enzyme activities, intestine and hepatopancreas histological structure. Aquaculture.

[31] Gasco L., Finke M., van Huis A. (2018). Can diets containing insects promote animal health?. J. Insects Food Feed.

[32] Rimoldi S., Terova G., Ascione C., Giannico R., Brambilla F. (2018). Next generation sequencing for gut microbiome characterization in rainbow trout (*Oncorhynchus mykiss*) fed animal by-product meals as an alternative to fishmeal protein sources. PLoS ONE.

[33] Purschke B., Meinlschmidt P., Horn C., Rieder O., Jäger H. (2018). Improvement of techno-functional properties of edible insect protein from migratory locust by enzymatic hydrolysis. Eur. Food Res. Technol..

[34] Spranghers T., Ottoboni M., Klootwijk C., Ovyn A., Deboosere S., De Meulenaer B., Michiels J., Eeckhout M., De Clercq P., De Smet S. (2017). Nutritional composition of black soldier fly (*Hermetia illucens*) prepupae reared on different organic waste substrates. J. Sci. Food Agric..

[35] Truzzi C., Giorgini E., Annibaldi A., Antonucci M., Illuminati S., Scarponi G., Riolo P., Isidoro N., Conti C., Zarantoniello M. (2020). Fatty acids profile of black soldier fly (*Hermetia illucens*): Influence of feeding substrate based on coffee-waste silverskin enriched with microalgae. Anim. Feed Sci. Technol..

[36] Oonincx D.G.A.B., Van Keulen P., Finke M.D., Baines F.M., Vermeulen M., Bosch G. (2018). Evidence of Vitamin D synthesis in insects exposed to UVb light. Sci. Rep..

[37] Hoffmann L., Rawski M., Nogales-Merida S., Mazurkiewicz J. (2020). Dietary inclusion of Tenebrio molitor meal in sea trout larvae rearing: Effects on fish growth performance, survival, condition, and GIT and liver enzymatic activity. Ann. Anim. Sci..

[38] Li Y., Kortner T.M., Chikwati E.M., Munang’andu H.M., Lock E.J., Krogdahl Å. (2019). Gut health and vaccination response in pre-smolt Atlantic salmon (Salmo salar) fed black soldier fly (Hermetia illucens) larvae meal. Fish Shellfish Immunol..

[39] Storebakken T. (2002). Atlantic salmon, *Salmo salar*. Nutrient Requirements and Feeding of Finfish for Aquaculture.

[40] Ministry of Science and Higher Education, Poland. http://www.bip.nauka.gov.pl/dobre-praktyki/.

[41] Leary S., Underwood W., Anthony R., Cartner S. (2013). AVMA Guidelines for the Euthanasia of Animals: 2013 Edition.

[42] AOAC (2005). Official Methods of Analysis of AOAC International.

[43] Soon C.Y., Tee Y.B., Tan C.H., Rosnita A.T., Khalina A. (2018). Extraction and physicochemical characterization of chitin and chitosan from *Zophobas morio* larvae in varying sodium hydroxide concentration. Int. J. Biol. Macromol..

[44] Kolodziejski P.A., Sassek M., Chalupka D., Leciejewska N., Nogowski L., Mackowiak P., Jozefiak D., Stadnicka K., Siwek M., Bednarczyk M. (2018). GLP1 and GIP are involved in the action of synbiotics in broiler chickens. J. Anim. Sci. Biotechnol..

[45] Kołodziejski P.A., Pruszyńska-Oszmałek E., Strowski M.Z., Nowak K.W. (2017). Long-term obestatin treatment of mice type 2 diabetes increases insulin sensitivity and improves liver function. Endocrine.

[46] Folch J., Lees M., Sloane Stanley G.H. (1957). A simple method for the isolation and purification of total lipides from animal tissues. J. Biol. Chem..

[47] Sobolewska A., Elminowska-Wenda G., Bogucka J., Dankowiakowska A., Kułakowska A., Szczerba A., Stadnicka K., Szpinda M., Bednarczyk M. (2017). The influence of in ovo injection with the prebiotic DiNovo® on the development of histomorphological parameters of the duodenum, body mass and productivity in large-scale poultry production conditions. J. Anim. Sci. Biotechnol..

[48] Giorgini E., Randazzo B., Gioacchini G., Cardinaletti G., Vaccari L., Tibaldi E., Olivotto I. (2018). New insights on the macromolecular building of rainbow trout (*O. mykiss*) intestine: FTIR Imaging and histological correlative study. Aquaculture.

[49] Rawski M., Kieronczyk B., Dlugosz J., Swiatkiewicz S., Józefiak D. (2016). Dietary probiotics affect gastrointestinal microbiota, histological structure and shell mineralization in turtles. PLoS ONE.

[50] Rawski M., Kierończyk B., Świątkiewicz S., Józefiak D. (2018). Long-term study on single and multiple species probiotic preparations for florida softshell turtle (*Apalone ferox*) nutrition. Anim. Sci. Pap. Rep..

[51] Huber I., Spanggaard B., Appel K.F., Rossen L., Nielsen T., Gram L. (2004). Phylogenetic analysis and in situ identification of the intestinal microbial community of rainbow trout (*Oncorhynchus mykiss*, Walbaum). J. Appl. Microbiol..

[52] Salzman N.H., de Jong H., Paterson Y., Harmsen H.J.M., Welling G.W., Bos N.A. (2002). Analysis of 16S libraries of mouse gastrointestinal microflora reveals a large new group of mouse intestinal bacteria. Microbiology.

[53] Waar K., Degener J.E., Van Luyn M.J., Harmsen H.J.M. (2005). Fluorescent in situ hybridization with specific DNA probes offers adequate detection of *Enterococcus faecalis* and *Enterococcus faecium* in clinical samples. J. Med. Microbiol..

[54] Sghir A., Gramet G., Suau A., Rochet V., Pochart P., Dore J. (2000). Quantification of bacterial groups within human fecal flora by oligonucleotide probe hybridization. Appl. Environ. Microbiol..

[55] Dong X.Q., Zhang D.M., Chen Y.K., Wang Q.J., Yang Y.Y. (2015). Effects of antimicrobial peptides (AMPs) on blood biochemical parameters, antioxidase activity, and immune function in the common carp (*Cyprinus carpio*). Fish Shellfish Immunol..

[56] Cha S.H., Lee J.S., Song C.B., Lee K.J., Jeon Y.J. (2008). Effects of chitosan-coated diet on improving water quality and innate immunity in the olive flounder, Paralichthys olivaceus. Aquaculture.

[57] Esteban M.A., Cuesta A., Ortuño J., Meseguer J. (2001). Immunomodulatory effects of dietary intake of chitin on gilthead seabream (*Sparus aurata* L.) innate immune system. Fish Shellfish Immunol..

[58] Yi H.Y., Chowdhury M., Huang Y.D., Yu X.Q. (2014). Insect antimicrobial peptides and their applications. Appl. Microbiol. Biotechnol..

[59] Kizak V., Guner Y., Turel M., Can E., Kayim M. (2011). Comparison of the survival and growth performance in rainbow trout (*Oncorhynchus mykiss*) and brown trout (*Salmo trutta fario*) fry. Afr. J. Agric. Res..

[60] Peruzzi S., Puvanendran V., Riesen G., Seim R.R., Hagen Ø., Martínez-Llorens S., Falk-Petersen I.B., Fernandes J.M.O., Jobling M. (2018). Growth and development of skeletal anomalies in diploid and triploid Atlantic salmon (*Salmo salar*) fed phosphorus-rich diets with fish meal and hydrolyzed fish protein. PLoS ONE.

[61] Andrews S.R., Sahu N.P., Pal A.K., Kumar S. (2009). Haematological modulation and growth of Labeo rohita fingerlings: Effect of dietary mannan oligosaccharide, yeast extract, protein hydrolysate and chlorella. Aquac. Res..

[62] Martínez-Alvarez O., Chamorro S., Brenes A. (2015). Protein hydrolysates from animal processing by-products as a source of bioactive molecules with interest in animal feeding: A review. Food Res. Int..

[63] Finke M.D. (2007). Estimate of chitin in raw whole insects. Zoo Biol..

[64] Janssen R.H., Vincken J.P., Van Den Broek L.A.M., Fogliano V., Lakemond C.M.M. (2017). Nitrogen-to-Protein Conversion Factors for Three Edible Insects: *Tenebrio molitor*, *Alphitobius diaperinus*, and *Hermetia illucens*. J. Agric. Food Chem..

[65] Xu H., Dong X., Zuo R., Mai K., Ai Q. (2016). Response of juvenile Japanese seabass (*Lateolabrax japonicus*) to different dietary fatty acid profiles: Growth performance, tissue lipid accumulation, liver histology and flesh texture. Aquaculture.

[66] Huang Y., Wen X., Li S., Li W., Zhu D. (2016). Effects of dietary lipid levels on growth, feed utilization, body composition, fatty acid profiles and antioxidant parameters of juvenile chu’s croaker Nibea coibor. Aquac. Int..

[67] Kowalska A., Zakęś Z., Siwicki A.K., Jankowska B., Jarmołowicz S., Demska-Zakęś K. (2012). Impact of Diets with Different Proportions of Linseed and Sunflower Oils on the Growth, Liver Histology, Immunological and Chemical Blood Parameters, and Proximate Composition of Pikeperch Sander Lucioperca (L.). Fish Physiol. Biochem..

[68] Piedecausa M.A., Mazón M.J., García García B., Hernández M.D. (2007). Effects of total replacement of fish oil by vegetable oils in the diets of sharpsnout seabream (*Diplodus puntazzo*). Aquaculture.

[69] Bhardwaj K., Verma N., Trivedi R.K., Bhardwaj S., Shukla N. (2016). Significance of ratio of Omega-3 and Omega-6 in human health with special reference to flaxseed oil. Int. J. Biol. Chem..

[70] Reddy P.B., Singh R.K. Biomarker responses in fish exposed to industrial effluent. Proceedings of the International Conference on Green Technology and Environmental Conservation, GTEC-2011.

[71] Loponte R., Nizza S., Bovera F., De Riu N., Fliegerova K., Lombardi P., Vassalotti G., Mastellone V., Nizza A., Moniello G. (2017). Growth performance, blood profiles and carcass traits of Barbary partridge (*Alectoris barbara*) fed two different insect larvae meals (*Tenebrio molitor* and *Hermetia illucens*). Res. Vet. Sci..

[72] Bovera F., Loponte R., Pero M.E., Cutrignelli M.I., Calabrò S., Musco N., Vassalotti G., Panettieri V., Lombardi P., Piccolo G. (2018). Laying performance, blood profiles, nutrient digestibility and inner organs traits of hens fed an insect meal from *Hermetia illucens* larvae. Res. Vet. Sci..

[73] Bovera F., Piccolo G., Gasco L., Marono S., Loponte R., Vassalotti G., Mastellone V., Lombardi P., Attia Y.A., Nizza A. (2015). Yellow mealworm larvae (*Tenebrio molitor*, L.) as a possible alternative to soybean meal in broiler diets. Br. Poult. Sci..

[74] Villanueva J., Vanacore R., Goicoechea O., Amthauer R. (1997). Intestinal alkaline phosphatase of the fish *Cyprinus carpio*: Regional distribution and membrane association. J. Exp. Zool..

[75] Agrahari S., Pandey K.C., Gopal K. (2007). Biochemical alteration induced by monocrotophos in the blood plasma of fish, *Channa punctatus* (Bloch). Pestic. Biochem. Physiol..

[76] Newsome P.N., Cramb R., Davison S.M., DIllon J.F., Foulerton M., Godfrey E.M., Hall R., Harrower U., Hudson M., Langford A. (2018). Guidelines on the management of abnormal liver blood tests. Gut.

[77] Lemieux H., Blier P., Dutil J.D. (1999). Do digestive enzymes set a physiological limit on growth rate and food conversion efficiency in the Atlantic cod (*Gadus morhua*)?. Fish Physiol. Biochem..

[78] McCarthy D.H., Stevenson J.P., Roberts M.S. (1973). Some blood parameters of the rainbow trout (*Salmo gairdneri* Richardson): I. The Kamloops variety. J. Fish Biol..

[79] Panettieri V., Chatzifotis S., Messina C.M., Olivotto I., Manuguerra S., Randazzo B., Ariano A., Bovera F., Santulli A., Severino L. (2020). Honey bee pollen in meagre (*Argyrosomus regius*) juvenile diets: Effects on growth, diet digestibility, intestinal traits, and biochemical markers related to health and stress. Animals.

[80] Gopal V., Parvathy S., Balasubramanian P.R. (1997). Effect of heavy metals on the blood protein biochemistry of the fish *Cyprinus carpio* and its use as a big-indicator of pollution stress. Environ. Monit. Assess..

[81] Sandnes K., Lie ø., Waagbø R. (1988). Normal ranges of some blood chemistry parameters in adult farmed Atlantic salmon, *Salmo salar*. J. Fish Biol..

[82] Aman Yaman M., Kita K., Okumura J. (2000). Different responses of protein synthesis to refeeding in various muscles of fasted chicks. Br. Poult. Sci..

[83] Belghit I., Liland N.S., Gjesdal P., Biancarosa I., Menchetti E., Li Y., Waagbø R., Krogdahl Å., Lock E.J. (2019). Black soldier fly larvae meal can replace fish meal in diets of sea-water phase Atlantic salmon (*Salmo salar*). Aquaculture.

[84] Zhou J.S., Liu S.S., Ji H., Yu H.B. (2018). Effect of replacing dietary fish meal with black soldier fly larvae meal on growth and fatty acid composition of Jian carp (*Cyprinus carpio* var. Jian). Aquac. Nutr..

[85] Cardinaletti G., Randazzo B., Messina M., Zarantoniello M., Giorgini E., Zimbelli A., Bruni L., Parisi G., Olivotto I., Tulli F. (2019). Effects of graded dietary inclusion level of full-fat *hermetia illucens* prepupae meal in practical diets for rainbow trout (*Oncorhynchus mykiss*). Animals.

[86] Khosravi S., Kim E., Lee Y.S., Lee S.M. (2018). Dietary inclusion of mealworm (*Tenebrio molitor*) meal as an alternative protein source in practical diets for juvenile rockfish (*Sebastes schlegeli*). Entomol. Res..

[87] Wang G., Peng K., Hu J., Yi C., Chen X., Wu H., Huang Y. (2019). Evaluation of defatted black soldier fly (*Hermetia illucens* L.) larvae meal as an alternative protein ingredient for juvenile Japanese seabass (*Lateolabrax japonicus*) diets. Aquaculture.

[88] Deng J., Kang B., Tao L., Rong H., Zhang X. (2013). Effects of dietary cholesterol on antioxidant capacity, non-specific immune response, and resistance to *Aeromonas hydrophila* in rainbow trout (*Oncorhynchus mykiss*) fed soybean meal-based diets. Fish Shellfish Immunol..

[89] Mashoof S., Criscitiello M.F. (2016). Fish immunoglobulins. Biology.

[90] Su J., Gong Y., Cao S., Lu F., Han D., Liu H., Jin J., Yang Y., Zhu X., Xie S. (2017). Effects of dietary *Tenebrio molitor* meal on the growth performance, immune response and disease resistance of yellow catfish (*Pelteobagrus fulvidraco*). Fish Shellfish Immunol..

[91] Józefiak A., Nogales-Mérida S., Rawski M., Kierończyk B., Mazurkiewicz J. (2019). Effects of insect diets on the gastrointestinal tract health and growth performance of Siberian sturgeon (*Acipenser baerii* Brandt, 1869). BMC Vet. Res..

[92] Bruni L., Pastorelli R., Viti C., Gasco L., Parisi G. (2018). Characterisation of the intestinal microbial communities of rainbow trout (*Oncorhynchus mykiss*) fed with *Hermetia illucens* (black soldier fly) partially defatted larva meal as partial dietary protein source. Aquaculture.

[93] Thankappan B., Ramesh D., Ramkumar S., Natarajaseenivasan K., Anbarasu K. (2015). Characterization of *Bacillus* spp. from the Gastrointestinal Tract of *Labeo rohita*—Towards to Identify Novel Probiotics against Fish Pathogens. Appl. Biochem. Biotechnol..

[94] Ramesh D., Vinothkanna A., Rai A.K., Vignesh V.S. (2015). Isolation of potential probiotic Bacillus spp. and assessment of their subcellular components to induce immune responses in *Labeo rohita* against *Aeromonas hydrophila*. Fish Shellfish Immunol..

[95] Antonopoulou E., Nikouli E., Piccolo G., Gasco L., Gai F., Chatzifotis S., Mente E., Kormas K.A. (2019). Reshaping gut bacterial communities after dietary *Tenebrio molitor* larvae meal supplementation in three fish species. Aquaculture.

[96] Ringø E., Wesmajervi M.S., Bendiksen H.R., Berg A., Olsen R.E., Johnsen T., Mikkelsen H., Seppola M., Strøm E., Holzapfel W. (2001). Identification and characterization of carnobacteria isolated from fish intestine. Syst. Appl. Microbiol..

[97] Karataş Düǧenci S., Candan A. (2003). Isolation of Aeromonas Strains from the Intestinal Flora of Atlantic Salmon (*Salmo salar* L. 1758). Turk. J. Vet. Anim. Sci..

[98] Ringø E., Jutfelt F., Kanapathippillai P., Bakken Y., Sundell K., Glette J., Mayhew T.M., Myklebust R., Olsen R.E. (2004). Damaging effect of the fish pathogen *Aeromonas salmonicida* ssp. salmonicida on intestinal enterocytes of Atlantic salmon (*Salmo salar* L.). Cell Tissue Res..

[99] Araújo C., Muñoz-Atienza E., Hernández P.E., Herranz C., Cintas L.M., Igrejas G., Poeta P. (2015). Evaluation of enterococcus spp. from rainbow trout (*Oncorhynchus mykiss*, Walbaum), feed, and rearing environment against fish pathogens. Foodborne Pathog. Dis..

[100] Osimani A., Milanović V., Roncolini A., Riolo P., Ruschioni S., Isidoro N., Loreto N., Franciosi E., Tuohy K., Olivotto I. (2019). Hermetia illucens in diets for zebrafish (*Danio rerio*): A study of bacterial diversity by using PCR-DGGE and metagenomic sequencing. PLoS ONE.

[101] Ringø E., Zhou Z., Olsen R.E., Song S.K. (2012). Use of chitin and, krill in aquaculture—The effect on gut microbiota and the immune system: A review. Aquac. Nutr..

[102] Askarian F., Zhou Z., Olsen R.E., Sperstad S., Ringø E. (2012). Culturable autochthonous gut bacteria in Atlantic salmon (*Salmo salar* L.) fed diets with or without chitin. Characterization by 16S rRNA gene sequencing, ability to produce enzymes and in vitro growth inhibition of four fish pathogens. Aquaculture.

[103] Tsai G.J., Su W.H., Chen H.C., Pan C.L. (2002). Antimicrobial activity of shrimp chitin and chitosan from different treatments and applications of fish preservation. Fish. Sci..

[104] Ringø E., Dimitroglou A., Hoseinifar S.H., Davies S.J. (2014). Prebiotics in Finfish: An Update. Aquaculture Nutrition.

[105] Song S.K., Beck B.R., Kim D., Park J., Kim J., Kim H.D., Ringø E. (2014). Prebiotics as immunostimulants in aquaculture: A review. Fish Shellfish Immunol..

[106] Zhou Z., Karlsen Ø., He S., Olsen R.E., Yao B., Ringø E. (2013). The effect of dietary chitin on the autochthonous gut bacteria of Atlantic cod (*Gadus morhua* L.). Aquac. Res..

